# Systemic Inflammation Mediates Age-Related Cognitive Deficits

**DOI:** 10.3389/fnagi.2018.00236

**Published:** 2018-08-06

**Authors:** Tian Lin, Gene A. Liu, Eliany Perez, Robert D. Rainer, Marcelo Febo, Yenisel Cruz-Almeida, Natalie C. Ebner

**Affiliations:** ^1^Department of Psychology, University of Florida, Gainesville, FL, United States; ^2^Department of Psychiatry, University of Florida, Gainesville, FL, United States; ^3^Pain Research and Intervention Center of Excellence, University of Florida, Gainesville, FL, United States; ^4^Center for Cognitive Aging and Memory, Department of Clinical and Health Psychology, University of Florida, Gainesville, FL, United States

**Keywords:** systemic inflammation, IL-6, cognitive aging, processing speed, moderated mediation

## Abstract

The association between systemic inflammation and cognitive deficits is well-documented. Further, previous studies have shown that systemic inflammation levels increase with age. The present study took a novel approach by examining the extent to which systemic inflammation levels mediated age-related cognitive decline. Forty-seven young and 46 older generally healthy adults completed two cognitive tasks measuring processing speed and short-term memory, respectively. Serum concentrations of three inflammatory biomarkers (including interleukin 6 (IL-6), tumor necrosis factor alpha (TNF-α), C-reactive protein (CRP)) were measured in each participant. Both cognitive measures showed age-related deficits. In addition, levels of IL-6 and TNF-α were elevated with age. IL-6 partially mediated the difference in processing speed between the young and the older participant age group; there was no mediation effect for TNF-α and CRP. Considering chronological age, IL-6 partially accounted for age-related impairment in processing speed within older but not young participants. No effects were found for short-term memory. Evidence from this research supports the role of inflammatory processes in age-related cognitive decline. Processes involved in this mediation and differences in inflammatory influence on specific cognitive functions are discussed.

## Introduction

### Systemic Inflammation and Cognitive Impairment

Mounting evidence associates cognitive impairment with systemic immune activation. For example, elevated serum levels of pro-inflammatory cytokines, including interleukin 6 (IL-6), tumor necrosis factor alpha (TNF-α) and C-reactive protein (CRP), lead to impairments in overall cognition (Weaver et al., 2002; Schram et al., 2007) as well as impairments in specific functions, such as reduced processing speed (Bettcher et al., 2014), executive function (Heringa et al., 2014) and memory (Teunissen et al., 2003). These associations between systemic inflammation and cognitive impairment have been found in young (Brydon et al., 2008), middle-aged (Marsland et al., 2008, 2015), and older (Athilingam et al., 2013; Tegeler et al., 2016) adults. Furthermore, within older adults, this inflammation−cognition link has been documented among generally healthy individuals (Brydon et al., 2008; Heringa et al., 2014; Nadkarni et al., 2016; Tegeler et al., 2016) and in clinical samples with conditions like dementia (Trollor et al., 2010), heart failure (Athilingam et al., 2013) and late-life depression (Charlton et al., 2018). However, to our knowledge, previous studies have not directly examined the mediatory role of systemic inflammation on cognitive aging.

Systemic inflammation leads to elevated circulating pro-inflammatory cytokines, including IL-6, TNF-α and CRP, which can interact with the central nervous system through three main routes: (i) pro-inflammatory cytokine transport proteins enable limited active transport across the blood brain barrier (BBB), allowing for central action (Dantzer et al., 2008; Fung et al., 2012). (ii) Systemically produced cytokines can stimulate afferent nerves (e.g., the vagal nerve), which then transmit the currently heightened inflammatory status to lower brain stem regions. In particular, the vagal nerve projects to the nucleus of the solitary tract and higher neural regions (e.g., hypothalamus, amygdala and bed nucleus of the stria terminals; McCusker and Kelley, 2013). (iii) Circulating cytokines reach the circumventricular organs, which reside outside the BBB. There, cells expressing toll-like receptors respond to the increased inflammatory state, eliciting further production and release of pro-inflammatory cytokines, which can then enter the brain through volume diffusion (Vitkovic et al., 2000; Dantzer et al., 2008; McCusker and Kelley, 2013; Sankowski et al., 2015).

These three pathways, when stimulated peripherally, will activate microglia and astrocytes in the brain to produce pro-inflammatory cytokines, propagating the signal into the neural environment (Dantzer et al., 2008; Sankowski et al., 2015). This leads to comparable inflammation levels in the brain and the periphery (McCusker and Kelley, 2013). Elevated neuroinflammation can result in structural and functional impairment in the brain (Varatharaj and Galea, 2017), such as hippocampal atrophy (Sankowski et al., 2015; Varatharaj and Galea, 2017) and increased substantia nigra activity (Brydon et al., 2008), both of which have been associated with cognitive deficits (Brydon et al., 2008; Sankowski et al., 2015; Varatharaj and Galea, 2017).

### Systemic Inflammation and Cognitive Aging

Pro-inflammatory cytokines can alter mitochondrial dynamics, decreasing metabolic efficiency and producing reactive oxygen species (ROS; Sankowski et al., 2015; van Horssen et al., 2017). ROS, in turn, can further elicit inflammatory immune response (Giunta, 2008; van Horssen et al., 2017), resulting in a positive feedforward loop. With accumulating oxidative stress and free radical damage, microglia and astrocytes become more likely to express a “primed” phenotype and morphology (Norden et al., 2015), characterized by an elevated baseline inflammatory state, a more vigorous pro-inflammatory response after reaching a lowered threshold, and resistance to any efforts to return to homeostasis (Norden and Godbout, 2013; Norden et al., 2015). The likelihood of progressing towards this primed phenotype increases with age as individuals are exposed to a greater amount of immune challenges. This increased risk for chronic low-grade inflammation in advanced age has been termed “inflammaging” (Giunta, 2008; Perry and Teeling, 2013; Dev et al., 2017; Charlton et al., 2018). A heightened inflammatory state, as seen with inflammaging, could partially explain the cognitive declines commonly observed in normal aging (Ownby, 2010).

Supporting this argument, recent research has reliably correlated increased inflammatory biomarker levels and diminished cognitive function in older adults (Bettcher et al., 2014; Heringa et al., 2014; Papenberg et al., 2016; Tegeler et al., 2016; Dev et al., 2017). These studies indicate that elevated systemic inflammation could be a risk factor for cognitive decline in old age, but do not directly imply that systemic inflammation mediates the age effect on cognitive functions. While some studies reported lower baseline cognitive scores in older adults with higher levels of systemic inflammation, these studies did not find evidence associating levels of systemic inflammation and rate of cognitive decline (Alley et al., 2008; Gimeno et al., 2008; Todd, 2017; but see Schram et al., 2007; Singh-Manoux et al., 2014). The present study, therefore, directly tested the mediation effect of systemic inflammation on age-related cognitive impairment in a sample with a wide age range across adulthood. One previous study examined the link between systemic inflammation and cognition in a sample with a relatively wide range (mean age = 65 years, SD = 11; Teunissen et al., 2003), but did not specifically test the mediation of systemic inflammation on cognitive aging.

### The Present Study

Adopting a novel methodological approach that considered between-group and within-group age effects, we expanded previous research in testing the role of systemic inflammation as a mediator of the age−cognition link in generally healthy adults. In particular, we addressed the following specific research questions: (i) Does systemic inflammation account for differences in cognitive performance between the young and the older age group? We predicted that greater systemic inflammation in the older compared to the young age group will mediate age-related cognitive impairment (i.e., reduced processing speed and short-term memory; *Hypothesis 1; mediation of the between-group age effect*). (ii) Does systemic inflammation account for age-related differences in cognitive performance within young and older age groups? We predicted that greater systemic inflammation associated with greater chronological age will mediate cognitive impairment in both the young and the older age group (*Hypothesis 2; mediation of the within-group age effect*). (iii) Does the extent to which systemic inflammation accounts for age-related differences in cognitive performance vary between young and older age groups? We predicted that the mediation of the within-group age effect will be more pronounced in the older compared to the young age group (*Hypothesis 3; moderated mediation of the within-group age effect*).

## Materials and Methods

### Participants

We recruited participants through: (i) handout and flyer distribution on campus and the community; (ii) HealthStreet, a university community outreach service; and (iii) mail-outs via two university participant registries particularly geared towards older individuals. Exclusion criteria were based on eligibility requirements for a larger project and included pregnancy (determined via pregnancy tests given to all women under 63 years), breastfeeding, psychological disorder, severe mental illness, excessive smoking or drinking and magnetic resonance imaging (MRI) incompatibility (as reported elsewhere; Ebner et al., 2015, 2016). The final sample for this report included 47 young (*M* = 22.3 years, *SD* = 2.73, 18–31 years, 47% female) and 46 older (*M* = 71.2 years, *SD* = 5.08, 63–80 years, 52% female) generally healthy adults.^1^ A basic metabolic panel, participants’ responses to a series of questions related to overall health, including diagnoses and medication intake, and a review of bodily systems by a licensed physician confirmed health status. All participants were Caucasian and fluent in English. As summarized in Table 1, older participants had more years of education than young participants. There was no difference between young and older participants in their self-reported physical and mental health.

**Table 1:** Means (SD)/median (Q1, Q3) and age-group differences in demographic, sleep, stress, health, cognitive and inflammation measures.

Construct	Young participants	Older participants
	(*n* = 47)	(*n* = 46)
Years of education	**15.46 (2.16)**	**16.62 (3.06)**
Typical sleep duration (in hours)	7.46 (1.45)	7.25 (1.09)
Current stress level	**3.35 (1.35)**	**2.44 (1.85)**
**Health**		
Number of bodily symptoms	**1 (0, 2)**	**4 (2, 7)**
Physical health	8.50 (1.13)	8.49 (1.00)
Mental health	8.46 (1.20)	8.84 (1.21)
**Cognitive measures**		
Processing speed	**64.38 (10.15)**	**45.72 (7.71)**
Short-term memory	**9.23 (1.94)**	**7.63 (2.44)**
**Inflammation markers**		
IL-6	**0.97 (0.71, 1.49)**	**2.07 (1.12, 2.62)**
TNF-α	**0.65 (0.45, 0.78)**	**0.91 (0.66, 1.25)**
CRP	613.7 (316.56, 1812.73)	957.82 (438.13, 2276.4)

*Note. Q1 = First Quartile; Q3 = Third Quartile. Typical Sleep Duration* “How many hours do you normally sleep per night?” *was measured by a single self-report item in hours. Current Stress Level* “Please rate your current stress” *was measured by a single self-report item on a scale from 1* = Not at all stressed *to 10* = Extremely stressed. *Number of Bodily Symptoms indicated the total number of symptoms that participants reported based on a brief review of all major bodily systems, in which participants indicated the presence (Yes or No) of each of 66 symptoms. Physical Health* “Please rate your general physical health” *and Mental Health* “Please rate your general mental health/mood” *were measured by single self-report items on a scale ranging from 1* = Poor *to 10* = Excellent. *We presented the median as well as the first and third quartiles for Number of Bodily Symptoms and for the inflammation biomarkers, given the positively skewed distribution of these variables. We applied bias-corrected bootstrapping with 10,000 resampling for the examination of age-group differences in the three inflammation biomarkers. Bold indicates significant age-group differences at p < 0.05*.

### Procedure

This data analysis was part of a larger project that comprised a phone screening and two campus visits. This report only includes data from the phone screening and the first campus visit (for additional publications from the larger project, see Ebner et al., 2015, 2016, 2018).

During the initial phone screening, older adults first underwent the Telephone Interview for Cognitive Status (TICS; *M* = 35.45, *SD* = 2.38, Min = 30, Max = 41, cut off ≤30; Brandt et al., 1988). All participants then completed the lab’s internal Health Screening and Demographics Form soliciting a range of information, including marital status, current medications, exercise habits and non-medicinal substance use such as caffeine, nicotine, alcohol and recreational drugs.

The phone screening was followed by a campus visit where, after receiving informed written consent, behavioral measures for short-term memory and processing speed (in this order; see details below) were administered. Next, a licensed physician conducted a review of all major bodily systems (i.e., eyes, ears, nose, throat; pulmonary; genito-urinary; gastrointestinal; neurological; cardiovascular; musculoskeletal) before a professional phlebotomist performed a blood draw to conduct the basic metabolic panel and to determine serum inflammation biomarker levels for IL-6, TNF-α and CRP. Personality and social relationship data were also collected, as reported elsewhere (Ebner et al., 2018). Participants were instructed to stay hydrated and avoid food, exercise and sexual activity for 2 h before the visit. They were also instructed to avoid smoking, caffeine, alcohol and recreational drugs for 24 h before the visit. Controlling for individual diurnal cycles, all screening visits began in the morning, usually around 8:00 a.m.

### Cognitive Measures

#### Processing Speed

The Digit Symbol-Substitution Task (DSST) from the Wechsler Adult Intelligence Scale (WAIS; Wechsler, 1981) was used to measure processing speed. In this task, participants are given a table pairing the digits 1 through 9 with nine distinct symbols (e.g., the number three is paired with a cross). Next, participants are presented with a sequence of 93 digits, ranging from 1 through 9. The goal is to identify individual digits, consider their corresponding symbol, and write that symbol in the space provided immediately below each respective digit, as quickly and accurately as possible. Participants are given 90 s to work through as much of the sequence as time permits. The number of correct responses was used to indicate processing speed, with higher scores indicating faster processing speed.

#### Short-Term Memory

The Rey Auditory Verbal Learning Test (RAVLT; Rey, 1964) was used to measure short-term memory. In this task, participants read a list of 15 words (letters per word: *M* = 5.0; word frequency: *M* = 55.8, *SD* = 86.3), one every 2 s, for concrete objects (e.g., “desk”, “ranger”). Immediately after, participants are asked to write down each word they can recall. The number of words correctly recalled was used to indicate short-term memory, with more recalled words indicating better short-term memory.

### Inflammation Biomarker Collection and Assay

Using serum tubes, a professional phlebotomist drew 10 mL of blood. After five inversions, samples rested at room temperature for 30–60 min to clot before 1600× *g* centrifugation at 4°C for 15 min and were immediately stored at −80°C. Before assays, samples thawed on ice. Using Human Quantikine Enzyme-linked Immunosorbent Assay (ELISA), serum IL-6, TNF-α and CRP levels were measured in duplicate according to instructions from the manufacturer (R and D System, Minneapolis, MA, USA; CRP: DCRP00; IL-6: HS600B; TNFα: HSTA00D). Intra-assay coefficient of variation (CV) was less than 6% for IL-6, 15% for TNF-α and 3% for CRP, and inter-assay CV was less than 14% for IL-6, 17% for TNF-α and 8% for CRP. IL-6 and TNF-α levels were significantly higher in older than young participants (Table 1). IL-6 levels were significantly related with CRP levels in both young and older participants and with TNF-α levels in older but not young participants. In contrast, TNF-α and CRP levels were not related in either of the age groups (Table 2).

**Table 2:** Bivariate correlations among the three inflammations markers in young and older participants.

	IL-6	TNF-α	CRP
IL-6		−0.03	**0.28**
TNF-α	**0.41**		0.18
CRP	**0.52**	0.34	

*Note. Correlation coefficients above the diagonal refer to young participants; correlation coefficients below the diagonal refer to older participants. We applied bias-corrected bootstrapping with 10,000 resampling to determine the significance of each correlation. Bold indicates significant age-group differences at p < 0.05. According to Fisher z-test, the correlation between interleukin 6 (IL-6) and tumor necrosis factor alpha (TNF-α) was significantly higher in older than young participants (z = −2.17, p = 0.03). In contrast, correlations between IL-6 and C-reactive protein (CRP; *z* = −1.35, p = 0.18) and between TNF-α and CRP (z = −0.80, p = 0.42) did not differ between the age groups*.

### Statistical Analysis

We conducted two separate analytic models to determine the extent to which systemic inflammation accounted for age effects on processing speed and short-term memory, respectively. In each model, the cognitive performance measure served as the dependent variable. Age-group contrast (categorical variable; young participants = −1; older participants = 1) and chronological age (continuous variable) served as the two independent variables in each model to capture both the between-group and the within-group age effects. To remove multicollinearity between the age-group contrast and the chronological age variables, we centered the chronological age variable separately in each age group. The three systemic inflammation biomarker levels served as mediators in the models. In addition, we considered the interaction between the age-group contrast and chronological age on the three systemic inflammation biomarkers and the cognitive performance measure and we considered the interaction between the age-group contrast and the three systemic inflammation biomarkers on the cognitive performance measure. This model specification allowed us to determine the extent to which the mediation of systemic inflammation of the within-group age effect on the cognitive measures varied between young and older participants (i.e., *moderated mediation of the within-group age effect*).

We used PROCESS macro on SPSS (Hayes, 2013) for model testing. The program reports the results for the mediation of the within-group age effects. However, it does not report the indirect effects of the between-group age effects. We followed guidelines by VanderWeele and Vanstellandt (VanderWeele and Vansteelandt, 2009; Valeri and VanderWeele, 2013) to calculate these indirect effects for each inflammation biomarker. We set significance thresholds of *p* < 0.05 (two-tailed test) for all main effects and interactions. We applied bias-corrected bootstrap with 10,000 resampling for calculation of the 95% confidence interval (CI) to determine the significance of each indirect effect.

## Results

### Mediation Effect of Systemic Inflammation on the Link Between Age and Processing Speed

#### IL-6

**Figure 1:**
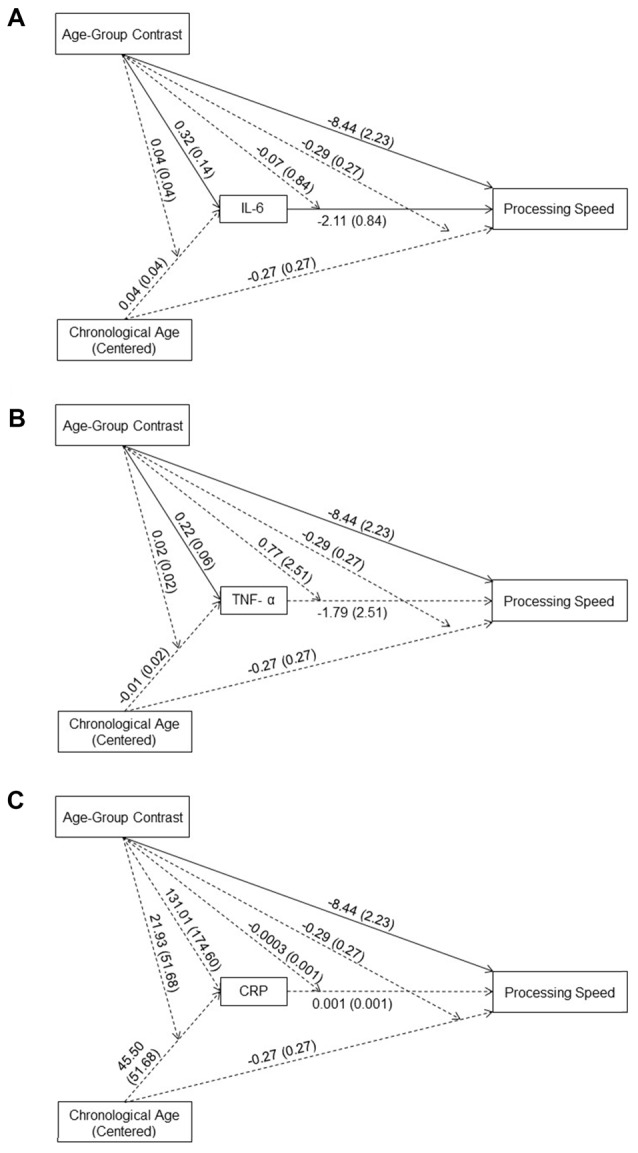
Effects (*B* (*SE*)) of chronological age (centered), age-group contrast and interleukin 6 (IL-6) **(A)**, tumor necrosis factor alpha (TNF-α) **(B)**, and C-reactive protein (CRP) **(C)**, and their interactions on processing speed. To improve readability, separate figures show the results from the three systemic inflammation biomarkers while all variables were considered in a single model. Solid lines indicate significance at *p* < 0.05.

The effect of age-group contrast on IL-6 was significant (*B* = 0.31, *t*_(89)_ = 2.22, *p* = 0.03) in the moderated mediation model (Figure 1A), with higher IL-6 levels in the older compared to the young group. In contrast, neither the main effect of chronological age (*B* = 0.04, *t*_(89)_ = 0.88, *p* = 0.38) nor its interaction with the age-group contrast (*B* = 0.04, *t*_(89)_ = 0.92, *p* = 0.36) was significant. That is, even though IL-6 levels were higher for individuals in the older compared to the young group, higher chronological age in each age group was not associated with higher IL-6 levels. The main effect of IL-6 levels on processing speed was significant (*B* = −2.11, *t*_(83)_ = −2.52, *p* = 0.01), while the interaction between IL-6 levels and age-group contrast was not significant (*B* = −0.07, *t*_(83)_ = −0.08, *p* = 0.93). That is, individuals with higher IL-6 levels had lower processing speed with comparable effects across the two age groups. Further, IL-6 levels mediated the between-group age effect on processing speed (Effect = −0.68, 95% CI = [−1.76, −0.06]) and the within-group age effect on processing speed in the older (Effect = −0.17, 95% CI = [−0.47, −0.02]) compared to the young (Effect = −0.003, 95% CI = [−0.27, 0.35]) group.

#### TNF-α

Similar to IL-6, the effect of age-group contrast on TNF-α was significant (*B* = 0.22, *t*_(89)_ = 3.68, *p* < 0.001) in the moderated mediation model (Figure 1B), with higher TNF-α levels in the older compared to the young group. In contrast, neither the main effect of chronological age (*B* = −0.01, *t*_(89)_ = −0.41, *p* = 0.68) nor its interaction with the age-group contrast (*B* = 0.02, *t*_(89)_ = 0.95, *p* = 0.34) on TNF-α levels was significant. That is, consistent with findings for IL-6, even though levels of TNF-α were higher for individuals in the older compared to the young group, higher chronological age in either age groups was not associated with higher TNF-α levels. Neither the main effect of TNF-α (*B* = −1.79, *t*_(83)_ = −0.71, *p* = 0.48) nor its interaction with age-group contrast (*B* = 0.77, *t*_(83)_ = 0.31, *p* = 0.76) on processing speed was significant, suggesting no association between TNF-α levels and processing speed in either age group. Furthermore, TNF-α levels did not mediate the between-group age effect on processing speed (Effect = −0.22, 95% CI = [−1.21, 0.31]) nor the within-group age effect in either the young (Effect = 0.06, 95% CI = [−0.16, 0.81]) or the older (Effect = −0.01, 95% CI = [−0.14, 0.03]) group.

#### CRP

The effect of age-group contrast on CRP was not significant (*B* = 131.01, *t*_(89)_ = 0.75, *p* = 0.46) in the moderated mediation model (Figure 1C), suggesting comparable CRP levels in the young and older groups. Also, neither the main effect of chronological age (*B* = 45.49, *t*_(89)_ = 0.88, *p* = 0.38) nor its interaction with the age-group contrast (*B* = 21.93, *t*_(89)_ = 0.42, *p* = 0.67) on CRP was significant, suggesting no age-related changes in CRP in either age group. In addition, neither the main effect of CRP (*B* = 0.001, *t*_(83)_ = 1.04, *p* = 0.30) nor its interaction with the age-group contrast (*B* = −0.0003, *t*_(83)_ = −0.50, *p* = 0.62) was significant, suggesting no association between CRP levels and processing speed in either age group. Also, CRP did not mediate the between-group age effect on processing speed (Effect = 0.04, 95% CI = [−0.11, 0.50]) or the within-group age effect on processing speed in either the young (Effect = 0.02, 95% CI = [−0.15, 0.36]) or older (Effect = 0.02, 95% CI = [−0.07, 0.23]) group.

#### Direct Age Effects After Controlling for the Effects of IL-6, TNF-α and CRP

In the moderated mediation model, the effect of age-group contrast on processing speed was significant (*B* = −8.44, *t*_(83)_ = −3.79, *p* < 0.001). That is, after accounting for the effect of the three biomarkers on processing speed, the older group still showed lower processing speed than the young group. In addition, neither the main effect of chronological age (*B* = −0.27, *t*_(83)_ = −0.99, *p* = 0.33) nor its interaction with the age-group contrast (*B* = −0.29, *t*_(83)_ = −1.06, *p* = 0.29) on processing speed was significant.

### Mediation Effect of Systemic Inflammation on the Link Between Age and Short-Term Memory

#### IL-6, TNF-α and CRP

**Figure 2:**
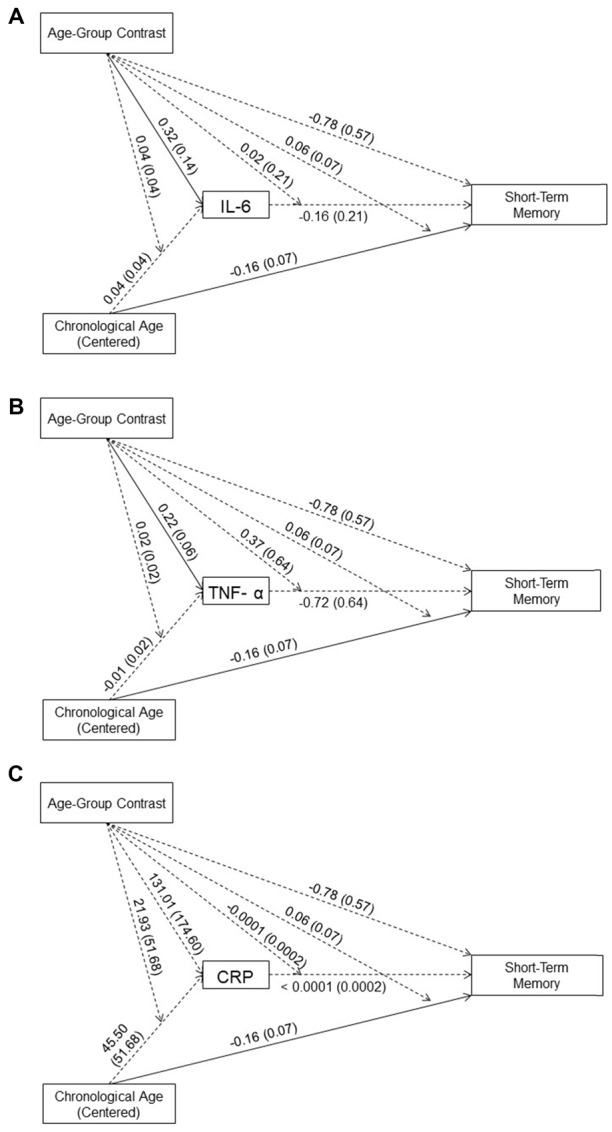
Effects (*B* (*SE*)) of chronological age (centered), age-group contrast, and IL-6 **(A)**, TNF-α **(B)**, and CRP **(C)**, and their interactions on short-term memory. To improve readability, separate figures show the results from the three systemic inflammation biomarkers while all variables were considered in a single model. Solid lines indicate significance at *p* < 0.05.

As shown in Figures 2A–C, neither the main effects of the three inflammation biomarkers nor their interaction with age-group contrast on short-term memory was significant. Thus, our data did not support an effect of systemic inflammation on short-term memory in either age group. In addition, none of the inflammation biomarkers mediated the between-group age effects nor the within-group age effects on short-term memory in the young or older age groups. These results indicated that inflammation biomarker levels did not account for age-related difference in short-term memory in our sample.

#### Direct Age Effects After Controlling for the Effects of IL-6, TNF-α and CRP

In the moderated mediation model, the effect of age-group contrast on short-term memory was not significant (*B* = −0.78, *t*_(83)_ = −1.37, *p* = 0.17). In contrast, the main effect of chronological age on short-term memory was significant (*B* = −0.16, *t*_(83)_ = −2.33, *p* = 0.02), while its interaction with age-group contrast was not significant (*B* = 0.06, *t*_(83)_ = 0.83, *p* = 0.41). That is, after accounting for the effect of the three inflammation markers on short-term memory, the age-related decline in short-term memory was comparable for the age groups^2^.

## Discussion

Going beyond previous work, the present study took a novel methodological approach by examining the mediation of systemic inflammation (i.e., serum levels of IL-6, TNF-α and CRP) on age-related cognitive impairments (i.e., deficits in processing speed and short-term memory). We found that systemic inflammation partially explained differences in cognitive performance associated with increased age. In particular, IL-6 levels accounted for the age-group difference in processing speed, supporting Hypothesis 1 (*mediation of the between-group age effect*). Further, IL-6 levels accounted for the age-related differences in processing speed within the older but not the young age group, supporting Hypothesis 3 (*moderated mediation of the within-group age effect*). However, our data did not support Hypothesis 2 (*mediation of the within-group age effect*). Neither of the remaining two examined inflammatory biomarkers (i.e., TNF-α, CRP) nor short-term memory yielded any significant effects. Of note, the sample size in the present study was relatively small, limiting the statistical power to detect small effects and calling for future replication of our findings in a larger sample. Next, we discuss the novel findings generated in this work and their implications in more detail.

### Age-Moderated Mediation of the Effect of Systemic Inflammation on Cognitive Aging

Previous work has documented a relationship between systemic inflammation and cognitive performance throughout adulthood, spanning young (Brydon et al., 2008), middle-aged (Marsland et al., 2008, 2015) and older (Athilingam et al., 2013; Tegeler et al., 2016) adults. The present study, however, represents the first direct test of a mediation effect of systemic inflammation on age-related cognitive impairment. In particular, our results suggest that the difference in systemic inflammation (measured by serum IL-6 levels) between young and older adults partially accounted for the difference in processing speed between these age groups. Additionally, IL-6 levels mediated age-related differences in processing speed within the older but not the young age group. Combining these two findings, the mediation of systemic inflammation on age-related variances in cognitive performance may become more pronounced with increased age (i.e., moderated mediation).

Two possible mechanisms may underlie the observed moderated mediation. First, age may increase the impact of systemic inflammation on cognition. In line with this suggestion, Baltes’s (1997) life-span developmental theory (see also Kwasnicka et al., 2016) proposes that biological factors become increasingly influential on behavior as older adults experience reduced functioning and less effective compensatory processes to counter functional decline. However, in the present study we did not find a significant age-moderation of the effect of IL-6 levels on processing speed (Figure 1A). That is, the association between IL-6 levels and processing speed was comparable between young and older adults. Similarly, a previous study showed that an experimentally-induced elevation in inflammatory cytokine response (i.e., typhoid vaccination) consistently hindered reaction times among young participants (Brydon et al., 2008). This suggests that systemic inflammation produces similar impairments regardless of individual age. Therefore, individuals with higher systemic inflammatory levels, regardless of age, are more likely to show cognitive impairments. Thus, our data combined with previous studies, does not support the age-related enhancement in the association between systemic inflammation and cognitive impairment as an explanation for the observed moderated mediation.

Second, systemic inflammation levels increase with age, possibly because older adults face more immune challenges and become increasingly likely to display mild chronic inflammation (inflammaging; Giunta, 2008; Perry and Teeling, 2013; Dev et al., 2017; Charlton et al., 2018). With chronic conditions, primed microglia can yield deleterious effects on their local neuro-environment, eliciting even greater inflammation, which may further prime microglia. This, in combination with continued accumulation of immune challenges, implies that inflammation levels, and their subsequent influence on cognition, may accelerate with time (Norden et al., 2015). Previous longitudinal studies, however, found no associations between systemic inflammation levels and the rate of cognitive decline (Alley et al., 2008; Gimeno et al., 2008; Todd, 2017). Importantly, these earlier studies focused on cohorts of older adults only. Further, while participants were tracked for about 10 year periods, this time span may have been too short to capture causal effects (Todd, 2017). Following from this argument, findings from the present study, which investigated a wider age range, showed that IL-6 levels partially accounted for the variance in processing speed between young and older adults. However, the cross-sectional nature of the present study does not allow causal conclusions of a mediation of inflammation on cognitive aging. Future longitudinal studies with longer data collection periods (e.g., across adulthood) are warranted.

### Domain-Specificity of the Mediation Effect of Systemic Inflammation on Cognitive Aging

While participants showed age-related cognitive impairments in both cognitive tasks, systemic inflammation only accounted for the age-related differences in processing speed but not short-term memory. In line with these findings, previous work had documented domain-specificity in the inflammation−cognition link. Heringa et al. (2014), for example, showed that older adults’ low-grade inflammation level, determined by the composite scores of various inflammatory biomarkers, including IL-6, IL-8, TNF-α and CRP, was negatively associated with processing speed, attention and executive functioning, but not other cognitive domains, including memory. Similarly, Tegeler et al. (2016) showed that higher levels of IL-6, IL-10 and CRP were associated with poorer executive function and processing speed, but not memory. In line with this correlational evidence, a recent intervention study found that participants who received antioxidant supplementation (e.g., resveratrol) for 90 days, compared to those in a control group who received placebo, showed improvement on processing speed (Anton et al., 2018; for a recent meta-analysis, also see Marx et al., 2018).

Importantly, microglial cells, which potentially represent the central mechanism for the neurological effects of inflammation, are widespread in the brain (Sankowski et al., 2015; van Horssen et al., 2017). This means that cognitive processes that integrate various areas across the brain may be more immediately vulnerable to inflammaging. Furthermore, a previous study reported a positive correlation between processing speed and whole-brain white matter volume, but not white matter volume from any sub-region in healthy young adults (Magistro et al., 2015). In addition, diffusion tensor imaging showed that processing speed in older adults was correlated with white matter integrity in diffuse areas of the frontal and parietal lobes (Kerchner et al., 2012). These results imply that processing speed is a cognitive process requiring coordination between various brain regions. Therefore, evidence from the present and previous studies associating systemic inflammation and processing speed, but not short-term memory (a more functionally localized process), supports the argument that systemic inflammation may cause global and diffuse brain damage with variable effects on individual cognitive domains.

We used the DSST to measure processing speed. Although the DSST has been commonly used as a measure of processing speed, previous research suggests that in addition to processing speed, other cognitive components such as executive function, visual scanning and memory contribute to performance in the DSST (Joy et al., 2003). Thus, participants’ performance in the DSST may not only reflect their processing speed but represent a broader cognitive functioning composite. Future studies could apply multiple cognitive tasks and adopt a latent factor approach to clarify the associations between systemic inflammation and various cognitive functions.

In contrast, Charlton et al. (2018) reported an association between elevated inflammatory cytokines (i.e., IL-6) and memory impairment in older adults with late-life depression, but not in a healthy control group. Importantly, cytokine measures in individuals with late-life depression were compared with measures in relatively healthy older adults. The study showed a significant correlation between inflammation biomarker levels and the severity of depressive symptoms. Thus, it is possible that psychological conditions, like depression, introduce additional inflammation in peripheral and central immune systems, enhancing the impact of inflammation on various neurological structures and functions. As a result, more localized cognitive domains (e.g., memory) could become more vulnerable in these clinical conditions. Consistent with this notion is evidence of an age-related decline in hippocampal sub-region volume in adults with hypertension, but not individuals with normal blood pressure (Bender et al., 2013). Further supporting this argument, previous studies suggest elevated systemic inflammation as a risk factor for cognitive impairment (e.g., dementia, Fung et al., 2012; Hoogland et al., 2015; Marsland et al., 2015) among older adults. Thus, it is possible that the present study’s exclusion criteria (e.g., no major illnesses, disorders, obesity), resulting in a sample of individuals with a relatively high health status, especially compared to older adults in the larger population, may have contributed to the lack of correlation between systemic inflammation and short-term memory.

The present study was embedded in the context of a larger project, which only included Caucasian individuals to avoid potential confounds in some of the central project outcomes. There is evidence, however, that racial minorities experience higher levels of inflammation (Paalani et al., 2011; Stepanikova et al., 2017), which may further contribute to age-related cognitive deficits within those populations (Windham et al., 2014; Goldstein et al., 2015). Thus, the lack of racial/ethnic diversity in the present sample limits the generalizability of our findings. Directly comparing racial/ethnic groups or analysis on a population-representative sample in future research will determine applicability of the present findings.

## Conclusion

The present study directly tested the mediatory role of systemic inflammation on age-related differences in two cognitive domains (i.e., processing speed, short-term memory). Our findings establish systemic inflammation as a potential mechanism underlying cognitive impairments in aging. These results highlight the importance of reducing inflammation to promote cognitive health. Preventive measures, like regular erobic exercise and medications to reduce inflammation, adopted across the entire lifespan, may prove particularly important to protect against cognitive decline, especially among older adults.

## Ethics Statement

This study was carried out in accordance with the recommendations of the Institutional Review Board at University of Florida. The protocol was approved by the Institutional Review Board at University of Florida. All subjects gave written informed consent in accordance with the Declaration of Helsinki.

## Author Contributions

TL conceptualized the study, collected and analyzed the data, and wrote the first draft of the manuscript. GL conceptualized the study, analyzed the data, and wrote the first draft of the manuscript. EP and RR assisted in article editing. MF and YC-A revised the final manuscript draft. NE conceptualized the study, supervised data collection and data analysis, and revised the manuscript.

## Conflict of Interest Statement

The authors declare that the research was conducted in the absence of any commercial or financial relationships that could be construed as a potential conflict of interest. The handling Editor declared a shared affiliation, though no other collaboration, with the authors.

## Footnotes

^1^The original sample included 105 participants. Four young and eight older participants had missing inflammation biomarker data, resulting in a final sample size of 93 participants for the present analysis.

^2^Given an age-group difference in years of education in the current sample (Table 1), we conducted parallel analyses in which we considered years of education as covariate. The pattern of findings did not change by adding this covariate.
